# Exploring the Links between Complex Post-Traumatic Stress Disorder and Psychotic-Like Experiences in Adolescents: A Gaussian and Bayesian Network Approach

**DOI:** 10.1192/j.eurpsy.2025.628

**Published:** 2025-08-26

**Authors:** T. B. Jannini, G. Terrone, L. Sideli, C. Niolu, G. Di Lorenzo, A. Schimmenti, R. Rossi

**Affiliations:** 1 Department of Experimental Medicine; 2 Department of Systems Medicine, Tor Vergata University of Rome; 3 Department of Human Sciences, LUMSA University of Rome, Rome; 4 Scienze dell’Uomo e della Società, Università degli Studi di Enna “Kore”, Enna, Italy

## Abstract

**Introduction:**

Complex post-traumatic stress disorder (cPTSD) is a recently recognized condition characterized by the combination of core PTSD symptoms—re-experiencing, avoidance, and hyperarousal—alongside disturbances in self-organization (DSO), which include affective dysregulation, negative self-concept, and interpersonal difficulties. Emerging evidence suggests that these complex symptom domains not only lead to greater functional impairment compared to PTSD but are also closely associated with the onset of psychotic-like experiences (PLEs). PLEs, which encompass subthreshold cognitive and perceptual anomalies such as hallucinations and delusional thinking, are increasingly viewed as critical indicators of vulnerability to more severe psychopathological outcomes. Given the developmental vulnerability during adolescence, the intersection of cPTSD and PLEs during this period is of significant concern, particularly as it may signal the emergence of broader psychopathological trajectories.

**Objectives:**

The primary aim is to identify the key cPTSD symptoms driving the comorbidity with PLEs in adolescents using two different network analysis approaches. We indeed seek to clarify what symptoms explain are modt central in the relatioship between PTSD/cPTSD and PLEs and elucidate the directionality of these symptom relationships. We finally aim to also explore whether gender moderates these associations within the network structures. We hypothesize that cPTSD symptoms are central in understanding the relationship between trauma and PLEs.

**Methods:**

A group of 1,010 late adolescents was assessed for post-traumatic symptoms and PLEs. The study involved estimating the Gaussian Graphical network structure for PTSD/cPTSD symptoms and PLEs, examining their bridge centrality indices. Subsequently, a Bayesian network analysis was conducted to create a directed acyclic graph (DAG). Gender was considered a moderator in both the Gaussian and Bayesian models.

**Results:**

Findings revealed that affect dysregulation, a domain of cPTSD, had the strongest connection with the PLEs cluster. The Bayesian network analysis showed a pathway linking cPTSD symptoms of worthlessness and interpersonal difficulties to PLEs symptoms like paranoia and social anxiety. Additionally, gender differences were observed in both network models.

**Image 1:**

**Figure:**
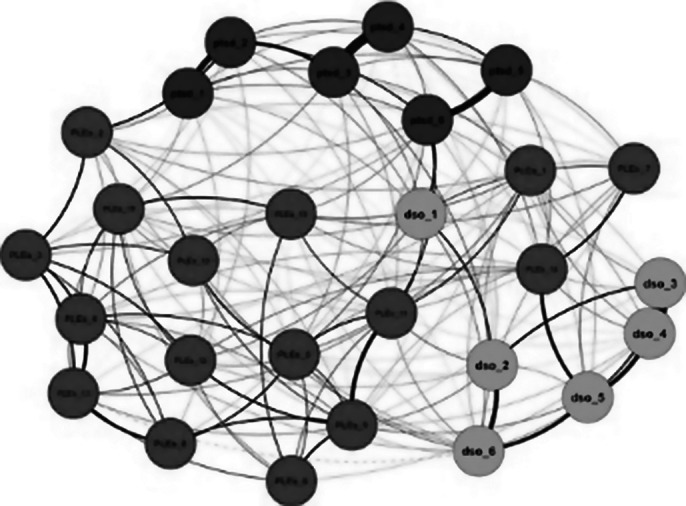

**Image 2:**

**Figure:**
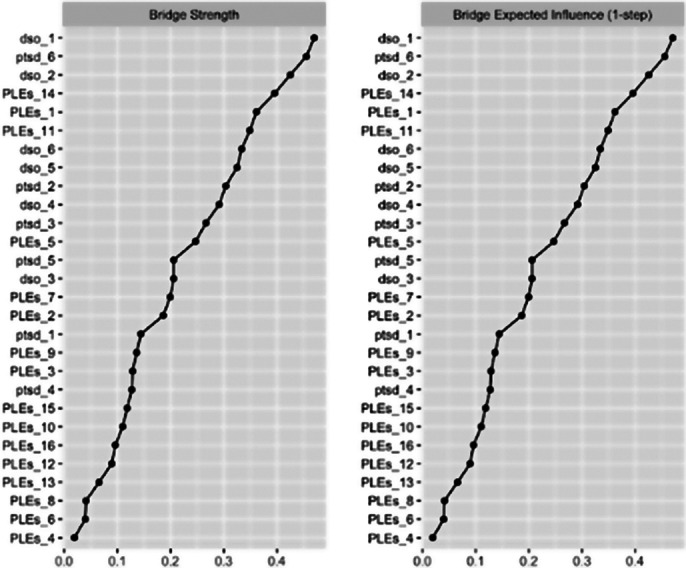

**Image 3:**

**Figure:**
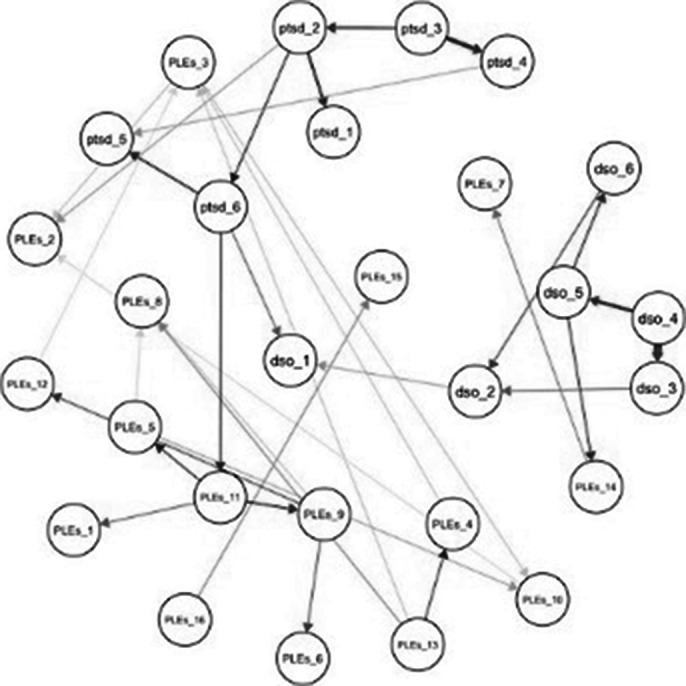

**Conclusions:**

The study emphasizes the pivotal role of affect dysregulation and a negative self-concept in associating cPTSD with PLEs, highlighting gender-specific differences. These results point to the importance of gender-sensitive strategies in preventing and treating PLEs in adolescents, stressing the need for early intervention and customized treatment plans.

**Disclosure of Interest:**

None Declared

